# Characterization and Bioactive Metabolite Profiling of *Streptomyces* sp. Y009: A Mangrove-Derived *Actinomycetia* with Anticancer and Antioxidant Potential

**DOI:** 10.3390/microorganisms12112300

**Published:** 2024-11-12

**Authors:** Bo Yu, Wei Zeng, Yuting Zhou, Nan Li, Zhiqun Liang

**Affiliations:** State Key Laboratory for Conservation and Utilization of Subtropical Agro-Bioresources, Guangxi Microorganism and Enzyme Research Center of Engineering Technology, College of Life Science and Technology, Guangxi University, 100 Daxue Road, Nanning 530004, China; 1908401010@st.gxu.edu.cn (B.Y.);

**Keywords:** *Streptomyces*, mangrove microorganisms, anticancer, antioxidant, oxidative stress, bioactive metabolites

## Abstract

Microorganisms from poorly explored environments are promising sources for the development of novel drugs. In our continuous efforts to screen for mangrove actinomycetes that produce metabolites with potential pharmaceutical applications, *Streptomyces* sp. Y009 was isolated from mangrove sediments in Guangxi, China. The phenotypic, physiological, biochemical, and phylogenetic characteristics of this strain were investigated. Analysis of phylogenetic and 16S rRNA gene sequences showed that it had the highest sequence similarity to *Streptomyces thermolilacinus* NBRC 14274 (98.95%). Further, the Y009 extract exhibited antioxidant activity, as indicated by DPPH and superoxide dismutase assays. The extract showed broad-spectrum and potent anticancer potential against six human cancer cell lines, with IC_50_ values ranging from 5.61 to 72.15 μg/mL. Furthermore, the selectivity index (SI) demonstrated that the Y009 extract exhibited less toxicity toward normal cell lines in comparison to the lung cancer cell line (A549) and hepatoma cell line (HepG2). GC–MS analysis revealed that the extract contained some biologically important secondary metabolites, mainly cyclic dipeptides and esters, which might be responsible for the antioxidant and anticancer properties. 3-Isobutylhexahydropyrrolo[1,2-a]pyrazine-1,4-dione (28.32%) was the major chemical compound available in the extract. The effect on cancer cells was then confirmed using nuclear staining and in silico docking. This study suggests that further exploration of the bioactive compounds of the newly isolated strain may be a promising approach for the development of novel chemopreventive drugs.

## 1. Introduction

Cancer seriously threatens human health, and the incidence and mortality rates are rapidly increasing worldwide [1]. The common cancers worldwide include lung, breast, colorectal, prostate, gastric, and liver cancers, accounting for approximately 50.7% of all cancers [2]. The current therapies are far from meeting the clinical needs, especially as existing drug therapies face the serious challenges of tumor resistance to chemotherapy and toxic side effects [3,4,5,6].

Thus, there is an urgent need to develop highly effective and low-toxicity anticancer drugs to meet the demand. Microorganisms are an essential source of lead compounds and innovative drugs. Recent years have seen an increase in research on the search for novel and efficient bioactive substances among secondary metabolites of microorganisms of different habitats [7,8,9,10]. Among these microorganisms, actinomycetes are a treasure trove for the discovery of a large number of medicinal natural products and occupy a very important position in the research on new microbial drugs [11,12,13,14]. Since the discovery of penicillin by Fleming, more than 33,500 bioactive secondary metabolites have been identified. Of these, about 13,000 secondary metabolites are derived from actinomycetes, involving 10,400 from *Streptomyces* and 3300 from other actinomycetes [15]. *Streptomyces* is widely present in the soil, sea, plant tissues, and air [16]. Natural secondary metabolites derived from *Streptomyces* account for 70–80% of the identified bioactive natural products that are employed in many fields, especially medicine, such as antibacterial, antifungal, anticancer, and antioxidant compounds, including enzymes and other bioactive substances. Doxorubicin, bleomycin, and epirubicin, which are produced by *S. peucetius*, *S. vertticillus,* and *S. nogalater* [17,18,19,20,21], are successfully used in the clinic to treat several types of cancer, such as lung cancer and breast cancer.

Mangroves are widely distributed in the transition area between the sea and land along tropical and subtropical coastlines [22]. Mangrove environments differ greatly from terrestrial environments. Its biodiversity far exceeds that of the land, and the unique natural environment has formed a unique metabolic pathway that distinguishes marine microorganisms from terrestrial ones, resulting in structurally diverse and biologically active metabolites and stimulating the evolution of new microorganisms. [23]. Therefore, there has been increasing interest in the exploitation of mangrove microorganism resources, leading to the discovery of novel *Streptomyces* species [24,25]. More than 20 new *Streptomyces* species have been discovered in mangrove and marine regions in recent years, such as *Streptomyces qinglanensis*, *Streptomyces mangrovisoli*, *Streptomyces malaysiense*, *Streptomyces marincola*, *Streptomyces monashensis*, and *Streptomyces qinzhouensis* [26,27,28,29,30,31]. Meanwhile, with the continuous research on natural products from mangrove *Streptomyces*, an increasing number of metabolites with novel structures and diverse activities are being discovered. Most of the structural types of these compounds are alkaloids, lactones, terpenoids, and peptides [32,33]. For instance, Streptocarbazoles A and B, two novel indolocarbazoles obtained from *Streptomyces* sp. FMA, showed cytotoxicity against A549 and HL-60 cell lines [34]. A new elaiophylin derivative, halichoblelide D, was isolated from mangrove-derived *Streptomyces* sp. 219807 and showed potent cytotoxicity against HeLa and MCF-7 cell lines [35]. This provides important resources for drug discovery and further proves the importance of mangrove *Streptomyces* in marine drug development.

This work is part of an ongoing project on the exploration of anticancer compounds from mangrove microbial resources. In our continuous efforts to investigate mangrove actinomycetes, *Streptomyces* sp. Y009 was isolated from sediments in the Guangxi mangrove forest, China. In vitro studies have revealed that the Y009 extract possesses noteworthy antioxidant and potent anticancer properties. The multi-target biological potential of Y009 in inducing anticancer and antioxidant effects was comprehensively demonstrated. Additionally, the bioactive chemical constituents of the extract have been further analyzed. Altogether, these results will provide important evidence for the further development of mangrove-derived *Streptomyces* metabolites as potential biopharmaceutical agents.

## 2. Materials and Methods

### 2.1. Strain Isolation and Maintenance

Strain Y009 was isolated from sediments from the subtropical mangrove nature reserve in Guangxi Province (China). Surface sediment samples (0–10 cm) were collected using the five-point sampling method, then evenly stirred into a sterile bag and stored at −20 °C. The samples were later suspended in sterile water (15 min at 50 °C) and diluted using tenfold serial dilution. On the isolation medium ZoBell 2216E, the dilutions were spread and incubated at 28 °C for 7 days [36]. For long-term preservation, purified cultures of strain Y009 were maintained on 2216E agar slants at 28 °C and as glycerol suspensions (30%, *v*/*v*) at −80 °C.

### 2.2. Genomic and Phylogenetic Analyses

Genomic DNA was extracted from strain Y009 using a genomic extraction kit (Sangon Biotech, Shanghai, China). 16S rRNA gene (16S rDNA) was amplified using the universal primers 27F (5′-AGAGTTTGATCMTGGCTCAG-3′) and 1492R (5′-TACGGYTACCTTGTTACGACTT-3′) [37]. The Clustal X v2.1 software was used to compare the 16S rRNA gene sequence of strain Y009 with representative sequences of *Streptomyces*-related types in the GenBank/EMBL/DDBJ database [38]. Based on the neighbor-joining and maximum-likelihood methods, the phylogenetic tree was constructed using MEGA v6.0 with 1000 repeats in a bootstrap analysis [39].

### 2.3. Culture and Phenotypic Properties

The culture characteristics of strain Y009 were determined following growth on ISP2, ISP4, ISP5, ISP7, Potato dextrose agar, Czapek’s agar, Gauze’s NO. 1 agar, Nutrient agar, and Bennett’s agar for 14 days at 28 °C. After 14 days of culture on various agars, the colors of the aerial mycelium, substrate mycelium, and soluble pigment produced by strain Y009 were determined using ISCC-NBS color charts [40]. A light microscope was used to observe the morphology of the strain after 7–14 days of incubation on ISP2 agar at 28 °C. The morphology of cells was also scanned using a scanning electron microscope (TM3000; Olympus, Tokyo, Japan) at 5000-fold magnification and a scanning voltage of 5 kV. The temperature range for growth was assessed on ISP2 agar with temperatures ranging from 4 °C to 45 °C. NaCl tolerance was assessed in tryptic soy broth (TSB) at 2% intervals, ranging from 0 to 12% (*w*/*v*). A pH range of 3.0 to 11.0 was investigated for growth in TSB. Temperature, pH, and NaCl responses were observed for 14 days [41]. Melanoid pigment production and catalase activity were determined using the protocols of Lee et al. [42]. The protease, cellulase, amylase, lipase, and chitinase activities of the strains were determined on ISP2 agar according to previously described methods [43]. Carbon source (glucose, sucrose, fructose, maltose, inulin, etc.) utilization was also studied.

### 2.4. Preparation of Y009 Fermented Broth and Extract

The strain was cultured in the fermentation medium containing 20 g/L inulin, 30 g/L peptone, and 1 g/L CaCl_2_. The medium was then sterilized (115 °C for 30 min), inoculated with Y009, and placed on a rotary shaker (180 rpm/min) for cultivation at 28 °C for 5 days. The broth was harvested, subjected to ultrasound to disrupt the cells, and centrifuged at 10,000× *g* for 10 min at 4 °C. Subsequently, the supernatant was mixed with D101 macroporous absorbent resin (5%) and stirred for 2 h. Thereafter, the resin was collected and washed extensively with 100% acetone. The eluate was then filtered and subjected to evaporation using a rotary vacuum evaporator at 40 °C. For further analysis, the final concentrated Y009 extract was suspended in dimethyl sulfoxide (DMSO) and stored at −20 °C.

### 2.5. Anticancer Activity

#### 2.5.1. Cell Culture

Human lung cancer cell line (A549), hepatoma cell line (HepG2), breast cancer cell line (MCF-7), colon cancer cell line (HCT116), nasopharyngeal carcinoma cell line (CNE-2), cervical cancer cell line (HeLa), and normal liver cell line (L-02) were routinely grown as monolayer cultures at 37 °C in a humidified atmosphere of 5% CO_2_ and 95% air. They were grown in Roswell Park Memorial Institute (RPMI)-1640 medium (Gibco) containing 10% (*v*/*v*) fetal bovine serum, 100 U/mL penicillin, and 100 μg/mL streptomycin (Solarbio).

#### 2.5.2. MTT Assay

Determination of the effect of *Streptomyces* sp. Y009 on the cell viability of human cancer cell lines using the established colorimetric MTT (3-(4,5-dimethylthiazol-2-yl)-2, 5-diphenyltetrazolium bromide) assay [44]. Briefly, 4.5 × 10^3^ cells/well were seeded in sterile flat-bottom 96-well plates (six replicate wells/group) and left to adhere overnight in an incubator. Next, Y009 extract suspended in DMSO was added into each well at various final concentrations, ranging from 2.5 to 40 µg/mL. The final concentration of DMSO did not exceed 0.1% (*v*/*v*). Conventional chemotherapeutic drug 5-fluorouracil was positive control. After 48 h of incubation with the extract, 20 μL of 5 mg/mL MTT was added to each well, and the plates were incubated at 37 °C in a humid atmosphere with 5% CO_2_ and 95% air for 4 h. After incubation, the MTT solution was removed carefully, and 150 μL of DMSO was added to dissolve the formazan. Microplate readers were used to measure the optical density of the solution at 490 nm. The percentage of cell viability was calculated by the following formula:

(1) $$\mathrm{Percentage}\;\mathrm{of}\;\mathrm{cell}\;\mathrm{viability}\;\%=\frac{\mathrm{Mean}\;\mathrm{absorbance}\;\mathrm{of}\;\mathrm{treated}\;\mathrm{cells}}{\mathrm{Mean}\;\mathrm{absorbance}\;\mathrm{of}\;\mathrm{untreated}\;\mathrm{cells}\;(0.1\%\;\mathrm{DMSO}\;\mathrm{only})}\times 100$$

The IC_50_ values were calculated using the IC_50_ online calculator (https://www.aatbio.com/tools/ic50-calculator) (accessed on 1 June 2023). Selectivity index: SI = IC_50_ value for normal cells/IC_50_ value for cancer cells.

#### 2.5.3. Hoechst 33342 Staining

Nuclear staining was detected using the Hoechst 33342 staining (Beyotime, Shanghai, China). The nuclear morphological features of untreated and treated A549 were examined under a fluorescence microscope.

### 2.6. Antioxidant Activity Assays

#### 2.6.1. Superoxide Anion Scavenging Activity Assay

The activity of superoxide dismutase (SOD) was assessed using a Total Superoxide Dismutase Assay Kit with WST-8 (Beyotime, Shanghai, China). A total of 20 µL of Y009 extract at various concentrations was added to a 96-well microplate and then mixed with 160 µL working solution and 20 µL reaction buffer according to the manufacturer’s instructions, using three parallel wells for each group. The total system was 200 µL; each mixture was incubated at 37 °C for 30 min, and the absorbance was detected at 450 nm.

#### 2.6.2. 2,2-Diphenyl-1-picrylhydrazyl (DPPH) Radical Scavenging Assay

DPPH radical scavenging activity was measured using a slightly modified protocol from a previous study [45]. A volume of 5 µL of Y009 extract in a series of concentrations was mixed with 195 µL of freshly prepared DPPH ethanol solution (0.4 mM) to make a final volume of 200 µL. DMSO was used as blank control, and the antioxidant 2,6-di-tert-butyl-4-methylphenol (BHT Macklin, Shanghai, China) was used as positive control. In this experiment, five parallel holes were used for each group. An ultraviolet-visible spectrophotometer was used to measure the reduction in DPPH radicals at 517 nm after the mixture was kept at room temperature for 20 min in the dark. The free radical-scavenging rate was calculated as follows:

(2) $$\mathrm{Inhibition}\%=\frac{\mathrm{Absorbance}\;\mathrm{of}\;\mathrm{control}-\mathrm{Absorbance}\;\mathrm{of}\;\mathrm{sample}}{\mathrm{Absorbance}\;\mathrm{of}\;\mathrm{control}}\times 100$$

### 2.7. Gas Chromatography–Mass Spectroscopy (GC–MS) Analysis

GC–MS analysis of the Y009 extract was performed using a Thermo Scientific TRACE 1300-TSQ 9000 with a TG-5SIMS capillary column (30.0 m × 250 µm inner diameter × 0.25 µm film thickness). The injection volume was 1 μL, and the sample was dissolved in 100% methanol (GC grade, Sigma-Aldrich, Burlington, MA, USA) at a concentration of 0.1 mg/mL. Helium was used as the carrier gas at a flow rate of 1 mL/min. The column temperature was initially at 40 °C for 5 min, then raised to 280 °C at a rate of 5 °C/min, and then kept isothermally at 280 °C for 5 min. The scan range was 30 to 600 *m*/*z*. Data from the NIST 05 Spectral Library were compared with mass spectral data of the extract constituents for identification.

### 2.8. Molecular Docking Analysis

The binding affinities and modes of interaction between the obtained small molecules and candidate targets were analyzed using AutodockVina 1.2.2. The molecular structure of 3-Isobutylhexahydropyrrolo[1,2-a]pyrazine-1,4-dione was retrieved from PubChem Compound (https://pubchem.ncbi.nlm.nih.gov/; accessed on 5 October 2023). The 3D coordinates of cyclin-dependent protein kinase 2 (CDK2, PDB ID, 3PXF; resolution, 2.5 Å) were downloaded from the PDB database. All protein and molecular files have been converted to PDBQT format with water molecules excluded and polar hydrogen atoms added for docking analysis. Protein binding sites were predicted using POCASA v1.1, and interaction pattern analysis of docking results was performed using PyMOL v2.3.0.

### 2.9. Statistical Analysis

All the assays on anticancer and antioxidant activities were conducted at least in triplicate, and data are expressed as mean ± standard deviation (SD). One-way analysis of variance (ANOVA) was used to determine the significance of the differences between the treated and control groups. Statistical analysis was performed using SPSS v24.0 software. Differences with *p* < 0.05 were considered statistically significant.

## 3. Results

### 3.1. Phenotypic Analyses of Strain Y009

**Figure 1:**
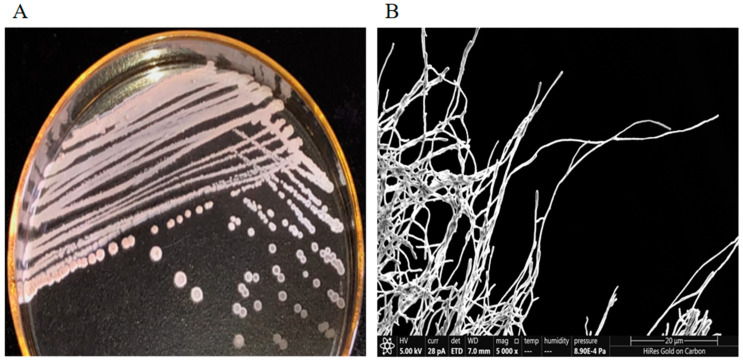
Physicochemical characteristics of *Streptomyces* sp. Y009. (**A**). The colony of Y009 cultured on an ISP2 medium. (**B**). Morphology of Y009 observed under a scanning electron microscope (SEM in the scale of 20 µm, magnification ×5000).

Strain Y009 exhibited good growth on ISP2 agar, ISP4 agar, Nutrient agar, Bennett’s agar, and Gauze’s agar and moderate growth on ISP5, ISP7, and PDA agar, whereas it grew poorly on Czapek’s agar (Supplementary Table S1). Combined with morphological observation and SEM (Figure 1A,B), it was found that the characteristics of aerial and vegetative hyphae were consistent with the morphological characteristics of the genus *Streptomyces* [46]. Growth of strain Y009 occurred at 24–37 °C (optimum 28 °C), NaCl tolerance ranges of 0–10% (optimum 8%), and pH ranges of 5.0–9.0 (optimum pH range 7–9.0) (Supplementary Figure S1). The Y009 cells were positive for melanoid pigment production but negative for H_2_S production. Positive results were obtained for the hydrolysis of soluble starch and cellulose. A variety of carbon sources can be utilized by strain Y009 (Table 1). The phenotypic properties could be used to differentiate strain Y009 from its closely related species of *Streptomyces* (Supplementary Table S2).

### 3.2. Phylogenetic and Genomic Analyses

**Figure 2:**
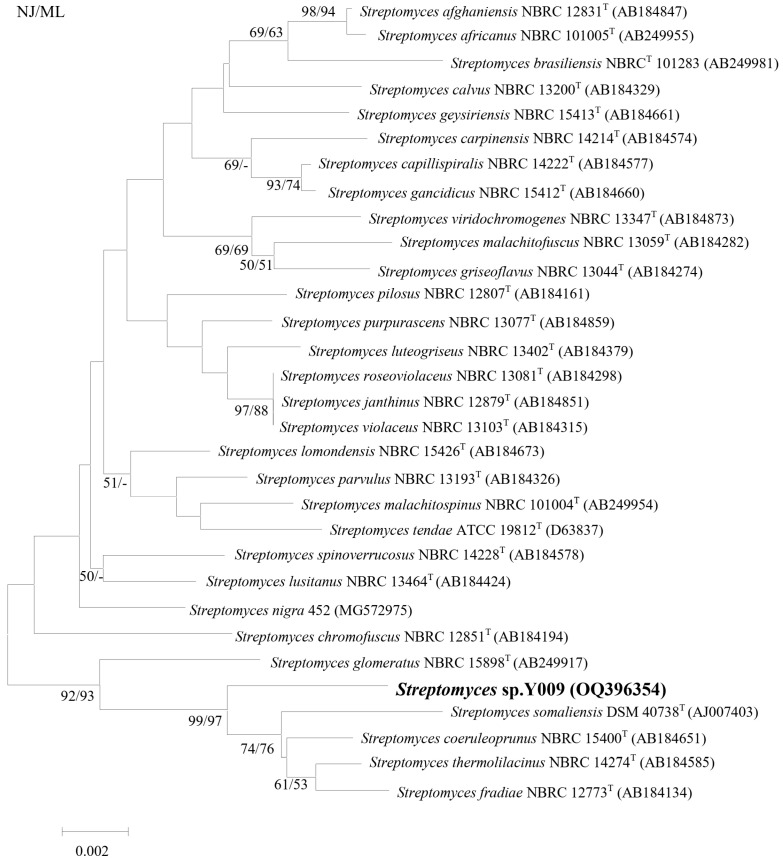
Neighbor-joining phylogenetic tree based on 16S rRNA gene sequences showing relationships between strain Y009 and related *Streptomyces* species. Numbers at nodes indicate percentages of 1000 bootstrap re-samplings, from left to right, for neighbor-joining and maximum-likelihood; only values above 50% are shown. Bar, 0.002 substitutions per site.

The nearly complete 16S rRNA gene sequences of strain Y009 were sequenced (GenBank No. OQ396354, 1430 bp). The phylogenetic tree based on 16S rRNA (Figure 2) showed that Y009 formed a distinct clade with type strains *Streptomyces thermolilacinus* NBRC 14274, *Streptomyces fradiae* NBRC 12773, *Streptomyces coeruleoprunus* NBRC 15400, and *Streptomyces somaliensis* DSM 40738 with a bootstrap value of 99/97%, showing the high confidence association value. The 16S rRNA sequence of strain Y009 exhibited closest similarity to *Streptomyces thermolilacinus* NBRC 14274 (98.95%), followed by *Streptomyces fradiae* NBRC 12773 (98.94%), *Streptomyces coeruleoprunus* NBRC 15400 (98.74%), and *Streptomyces somaliensis* DSM 40738 (98.45%).

### 3.3. Antioxidant Activity of Y009 Extract

The antioxidant activity of the Y009 extract was determined by assessing its radical scavenging of both superoxide anions and DPPH radicals (Table 2). At both 0.6 and 1.2 mg/mL, the Y009 extract significantly inhibited DPPH radicals (14.72 ± 2.6% and 24.30 ± 3.34%, respectively; *p* < 0.05). The inhibition of superoxide anions (47.48 ± 1.79% at the highest Y009 extract concentration of 1.2 mg/mL) confirmed the antioxidant potential of the Y009 extract.

### 3.4. Anticancer Activity of Y009 Extract

**Figure 3:**
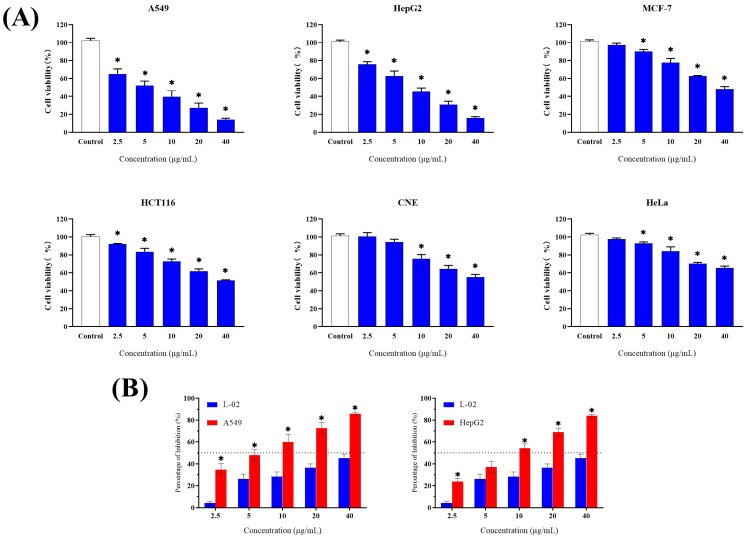
Cytotoxic activity of Y009 extract on human normal and cancer cell lines compared by MTT assay. (**A**) Cytotoxic effect of Y009 extract against human cancer cell lines. Each bar represents the mean of the cell viability of the cell lines after treatment with extract at respective concentrations tested (*n* = 6). The vertical lines associated with the bars represent the standard deviation of the mean. Symbol (*) indicates *p*-values < 0.05 significant difference compared to control. (**B**) Comparison of inhibition percentage of extract between normal cell line (L-02) and two cancer cell lines (A549, HepG2). (*n* = 6, * *p* < 0.05).

Human lung cancer (A549), hepatoma (HepG2), breast cancer (MCF-7), colon cancer (HCT116), nasopharyngeal carcinoma (CNE-2), and cervical cancer (HeLa) cell lines were used to evaluate the extract for cytotoxic activity based on MTT assays. In addition, the human normal liver cell line (L-02) was used to determine the toxicity of the extract against non-cancerous cells. Figure 3 shows the inhibitory effect of Y009 extract on cell viability after 48 h of treatment with various concentrations of the extract on each cell line. In detail, the Y009 extract inhibited the growth of six cancer cell lines to varying degrees (Figure 3A). All cancer cell lines were significantly inhibited by Y009 extract at medium concentrations (10 µg/mL) compared to the controls (*p* < 0.05). More importantly, four cell types were inhibited by ≥50% at the highest tested concentration (40 µg/mL). The A549 cell line was the most sensitive to treatment with the extract (IC_50_ of 5.614 µg/mL), followed by HepG2 (IC_50_ of 8.377 µg/mL) (Table 3), which was much lower than the reference drug (5-FU IC_50_ = 10.42 µg/mL and IC_50_ = 12.11 µg/mL, respectively). The IC_50_ of the extract against normal cells was calculated as 47.95 µg/mL (5-FU IC_50_ = 8.50 µg/mL). It is noteworthy that the extract has a higher level of toxicity against some cancer cells and a lower level of cytotoxicity against normal cell lines (Figure 3B). The inhibition percentage of the extract on normal cells was significantly lower than on A549 and HepG2 cells at the same concentration. And this is exemplified by the results of the selectivity index, compared to A549 cells with an SI value of 8.54, followed by HepG2 with an SI value of 5.72. Accordingly, the SI values of 5-FU are 0.82 and 0.70, respectively.

**Figure 4:**
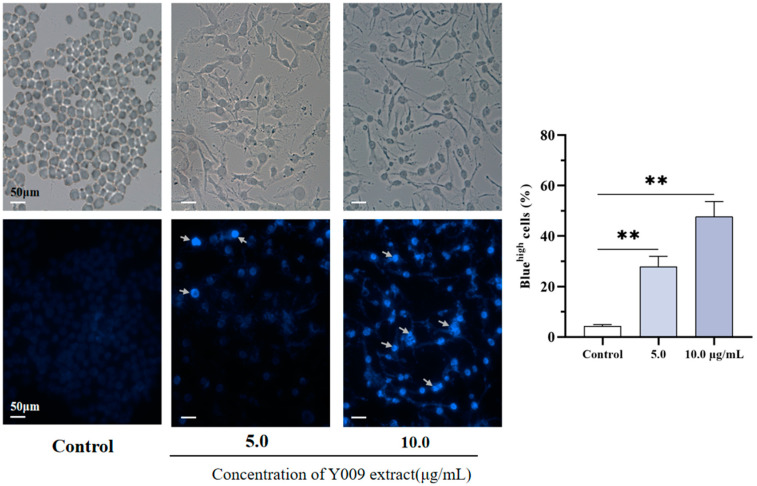
Morphology of A549 after treatment with Y009 extract. Comparison of the morphological features of A549 after 48 h with Y009 extract at respective concentrations observed under phase contrast microscopy. Nucleus counterstained with Hoechst 33342 (blue) under fluorescence microscope. A total of 300 nuclei were examined under the microscope in 5–8 randomly selected fields of view for quantification of nuclear Hoechst 33342 staining. Symbol (**) indicates *p*-values < 0.05 significant difference compared to control.

In addition, A549 cells showed morphological changes during 48 h of treatment with the extract. The control A549 cells had normal angular and spindle shapes after treatment with the extract; most cells lost these characteristics, and cells were elongated and more irregular in shape. In addition, there was a reduced number of cells and a loss of adhesion and cytoplasm mass. Likewise, Hoechst 33342 staining showed a difference between the treated and untreated A549 cell nuclei (Figure 4). In morphologically normal nuclei, Hoechst 33342 stains them dimly blue, whereas in treated cells, the dye stains bright blue due to nuclear atrophy or condensation and nuclear fragmentation (indicated by arrows). The percentage of bright blue cells was significantly higher than the control cells after the extract treatment. These abnormal cell morphological changes following exposure to Y009 extract provide insight into its cytotoxicity against A549 cells.

### 3.5. GC–MS Analysis of Y009 Extract

**Figure 5:**
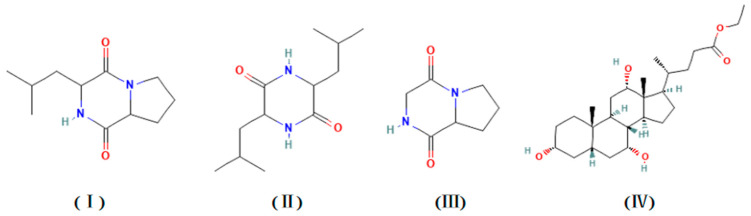
Chemical structures of the major metabolites obtained from Y009: three diketopiperazines (I–III) and an ester (IV). (**I**): 3-Isobutylhexahydropyrrolo[1,2-a]pyrazine-1,4-dione (28.32%); (**II**): 2,5-Piperazinedione,3,6-bis(2-methylpropyl)- (10.35%); (**III**): Hexahydropyrrolo[1,2-A]Pyrazine-1,4-Dione (9.87%); (**IV**): Ethyl iso-allocholate (9.48%).

GC–MS analysis identified the following 15 different compounds in the Y009 extract (Table 4). Based on the chromatogram peak percent area indicated the major compounds as (Supplement Figure S2) 3-Isobutylhexahydropyrrolo[1,2-a]pyrazine-1,4-dione (28.32%), 2,5-Piperazinedione,3,6-bis(2-methylpropyl)- (10.35%), Hexahydropyrrolo[1,2-A]Pyrazine-1,4-Dione (9.87%), Ethyl iso-allocholate (9.48%). (Chemical structures are shown in Figure 5).

### 3.6. Molecular Docking and Cytotoxic Activity of the Main Constituents on Cancer Cells Detected by GC–MS

**Figure 6:**
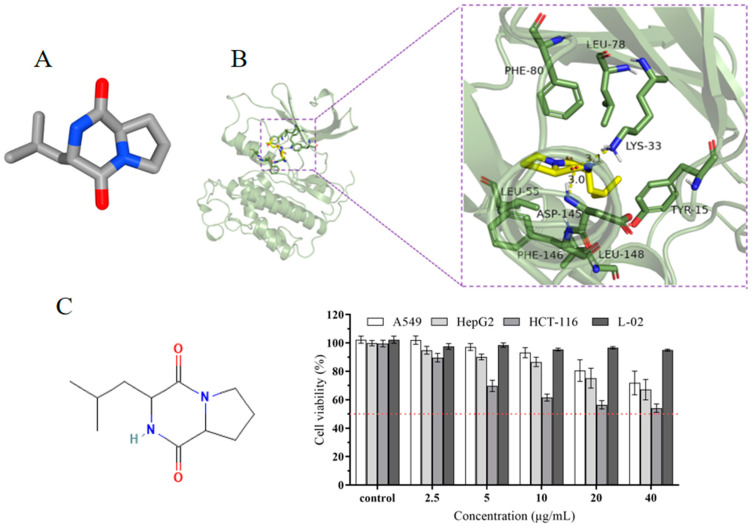
(**A**) Three-dimensional structures of 3-Isobutylhexahydropyrrolo[1,2-a]pyrazine-1,4-dione. (**B**) Three-dimensional crystal structure with selected compound and protein CDK2 complex. (**C**) Cytotoxic effects of 3-Isobutylhexahydropyrrolo[1,2-a]pyrazine-1,4-dione against human cancer cell lines and normal cells.

To further investigate the anticancer properties of the chemical compounds in the extract, we performed a molecular docking analysis to evaluate the affinity of the four leading bioactive compounds detected in the extracts, namely, 3-Isobutylhexahydropyrrolo[1,2-a]pyrazine-1,4-dione (compound **I**), 2,5-Piperazinedione,3,6-bis(2-methylpropyl)- (compound **II**), and Hexahydropyrrolo[1,2-A]Pyrazine-1,4-Dione (compound **III** and Ethyl iso-allocholate (compound **IV**), with cell cycle protein-dependent protein kinase 2 (CDK2). CDK2 is an attractive target for cancer therapy due to its key role in cell cycle progression. The binding energy values of the four compounds with CDK2 binding proteins were −7.2 kcal/mol for compound **I**, −6.2 kcal/mol for compound **II**, −5.7 kcal/mol for compound **III,** and −6.3 kcal/mol for compound **IV** (Supplement Table S3). Previous studies have reported that the lower the binding energy value, the stronger the binding to the target protein, with binding energy below −7.0 kcal/mol indicating very firm binding. The results show that the compound **I** bounds to the protein target through visible hydrogen bonding and hydrophobic interaction, forming hydrogen bonds with LYS-33 and ASP-145 with hydrogen bond lengths of 3.1 Å and 3.0 Å, respectively. It is a hydrophobic interaction with LEU-55, PHE-146, LEU-148, TYR-15, LEU-78, and PHE-80 and has a low binding energy of −7.2 kcal/mol, indicating highly stable binding (Figure 6A,B). In addition, three tumor cells were chosen for further investigation of the cytotoxic effect of compound **I**. The results showed that compound **I** exhibited different levels of in vitro cytotoxicity, with a dose-dependent effect observed. At the tested concentrations of 20 µg/mL and 40 µg/mL, a significant effect on the cell viability was observed across three types of cancer cell lines, with HCT-116 cells being the most sensitive to the treatment and the cell viability decreasing to 54% at 40 µg/mL. Interestingly, the compound did not exhibit significant cytotoxicity against normal L-02 cells at all the concentrations tested after 48 h of exposure (Figure 6C).

## 4. Discussion

Microorganisms have been an important source of drug development and are still an important source of natural products with novel structures. It has become increasingly difficult to isolate new actinomycete species from common habitats and to explore new bioactive products. However, scientists have achieved positive results regarding developing new natural products by using fermentation of actinomycetes from special habitats or using rare actinomycetes based on the “new environment, new strain, new product” strategy [23]. A mangrove ecosystem is a special environment, and the actinomycetes in these habitats have formed rich microbial groups and unique metabolic pathways through long-term natural processes of selection and evolutionary adaptation. Their metabolites have various structures and biological activities (antibacterial, anti-inflammatory, antitumor, antioxidant) that may differ from those of terrestrial actinomycetes. The metabolites from mangrove ecosystems provide valuable microbial resources for the search for new drugs [47].

In the present study, *Streptomyces* sp. Y009 was isolated from sediments in the Guangxi mangrove forest, China. Phylogenetic and morphological results indicate that Y009 belongs to the genus *Streptomyces*. Furthermore, it demonstrates the highest similarity (based on 16S rDNA) to *S. thermolilacinus* NBRC 14274 (98.95%), followed by *S. fradiae* NBRC 12773 (98.94%) and *S. somaliensis* DSM 40738 (98.45%). Kim et al. stated that 16S rRNA gene sequence similarity (<98.65%) could be used as a threshold to differentiate the two species [48]. This will, of course, need to be further analyzed and validated by whole genome sequencing, DNA-DNA hybridization, and average nucleotide identity to increase the accuracy of the results. In addition, we found no other reports in the literature on the antioxidant and anticancer activity of S. *thermolilacinus* NBRC 14274. This confirms the value of this study of metabolites of biological activity by strain Y009.

Oxidative stress is a phenomenon in which the body produces a large number of free radicals after being stimulated by external factors, which leads to cell and tissue damage. Oxidative stress is involved in the development of various multifactor diseases such as cancer, neurodegenerative diseases, and cardiovascular diseases [49]. Therefore, it is of great significance to identify antioxidants that can effectively eliminate free radicals for the treatment of diseases related to oxidative stress. Identifying antioxidant lead compounds based on natural products and carrying out structural modification and optimization are important ways to develop novel, effective drugs for treating oxidative stress-related diseases. Extensive research has shown that a number of potent antioxidative chemical compounds can be derived from microbes [41,50,51]. Additionally, high antioxidant capacity was found in microbes from extreme environments.

The DPPH radical scavenging assay is widely applied to evaluate the antioxidant activity of plant compounds and extracts of microbial origin. Discoloration occurs when the odd electron of the DPPH radical is paired off with antioxidants. The degree of color change (from a solution of purple DPPH radicals to yellowish–brown diphenylpicrylhydrazine) is positively correlated with the antioxidant capacity of the antioxidant reagent. In the present study, the DPPH assay indicated that the Y009 extract had significant antioxidant activity.

Furthermore, the dose-dependent superoxide anion scavenging activity further confirmed the antioxidant potential of the Y009 extract. Superoxide anions (O_2_^.−^) are the product of oxygen molecules, each reduced by a single electron. They are closely linked to biological processes, such as aging, inflammation, cancer, and exogenous metabolism. Decreasing them can reduce the damage to DNA [52]. In the current study, by comparing the amount of water-soluble formazan dye production caused by the reaction of WST-8 with superoxide anions, the Y009 extract was shown to have strong superoxide anion scavenging activity. In summary, the DPPH radical scavenging assay and superoxide anion scavenging activity assay revealed the significant antioxidant activity of the Y009 extract. This indicates the presence of valuable compound(s) in the Y009 extract, which may potentially reduce the occurrence of cancer and be further developed for chemopreventive treatment.

The MTT assays are widely employed in cytotoxicity evaluations to assess drug efficiency. MTT can be reduced by mitochondrial dehydrogenases to produce a crystalline dark purple product known as formazan, which can be completely soluble in the presence of specific solvents [53]. The Y009 extract had a significant dose-dependent inhibitory effect on the proliferation of human cancer cell lines tested, especially on lung cancer (A549) and hepatoma (HepG2) cells, and, importantly, it has lower cytotoxicity compared to normal cell lines. In addition, when comparing the cytotoxic activity of extracts from other sources [54,55,56], the Y009 extract has a lower IC_50_ value against A549 and HepG2 cells. Lung cancer is currently the leading cause of death from all cancers, and the search for efficient lung cancer therapeutic drugs is of great significance [2]. Broad spectrum, high efficiency, and selectivity are key characteristics of clinical chemotherapeutic drugs, and the Y009 extract has certain clinical research value and application prospects based on the in vitro anticancer results. Based on morphological changes assessed by phase contrast microscopy and Hoechst 33342 nuclear staining, the Y009 extract may exert its cytotoxic effects through a mechanism associated with apoptotic cell death, leading to decreased viability of cancer cells. This is supported by the observations of the cell morphology elongated, condensed, and fragmented chromatin, segregation of nucleoli, and diminished refractoriness observed in the Y009 extract group, which are typical morphological characteristics of apoptotic cells [57]. However, the mode of cell death needs to be determined by further studies on the molecular basis.

Prompted by the excellent antioxidant activity and in vitro antitumor activity exhibited by the Y009 extract, the chemical composition of the extract was ascertained. GC–MS analysis, which plays an important role in the discovery of natural products, including bioactive compounds derived from *Streptomyces* species [9,20,50], showed that the extract contained about fifteen compounds; some of the pharmacologically active compounds detected in this study have previously been detected in marine-derived microbial fermentation broth or extracts [58,59,60]; esters (12.98%) and several cyclic dipeptides (50.44%) were the main classes of compounds in the Y009 extract. Four of the compounds detected in this study, i.e., Hexahydropyrrolo[1,2-A]Pyrazine-1,4-Dione (9.87%), Cyclo(L-Pro-L-Val) (1.90%), 2,5-Piperazinedione,3,6-bis(2-methylpropyl)- (10.35%), and 3-Isobutylhexahydropyrrolo[1,2-a]pyrazine-1,4-dione (28.32%) belong to a group of cyclic dipeptides or 2,5-diketopiperazines (DKPs), which are a class of heterocyclic alkaloids widely found in nature. Their core backbone structure is a cyclic dipeptide formed by the condensation of two amino acids through a peptide bond. The conformationally constrained six-membered ring makes cyclic dipeptides pharmacodynamically attractive in medicinal chemistry, with a variety of bioactivities and pharmacological activities being reported [61,62]. For example, Hexahydropyrrolo[1,2-A]Pyrazine-1,4-Dione compound was detected in *Streptomyces mangrovisoli*, a new species of *Streptomyces* isolated from mangroves in Malaysia, which exhibited antioxidant activity [27]. A previous study reported that Cyclo(L-Pro-L-Val) extracted from *Streptomyces nigra* sp. had IC_50_ values of 67.2 and 102.9 µg/mL in cytotoxicity assays of colon cancer (HCT116) and hepatoma (HepG2) cells, respectively [20]. Similarly, 3-Isobutylhexahydropyrrolo[1,2-a]pyrazine-1,4-dione has been shown to have a selective cytotoxic effect and induced cell cycle arrest and apoptosis in colon cancer cells [9]. It has also been isolated from *Pseudonocardia endophutica* VUK-10 and has potent cytotoxic activity against breast cancer cell lines [63]. In addition, 2,5-Piperazinedione,3,6-bis(2-methylpropyl)-, a natural product found in *Bacillus subtilis*, has antibacterial activity [64]. Furthermore, Hexadecanoic acid, methyl ester (3.50%), and Ethyl iso-allocholate (9.48%) have been detected in a variety of sources, from plant extracts to endophytic fungus, but not detected in *streptomyces* spp. The newly isolated strain Y009, therefore, provides a fresh resource for the synthesis of these substances. Previous research reported that Hexadecanoic acid, methyl ester, possesses anti-inflammatory and antioxidant properties [65]. The cytotoxic compound Ethyl iso-allocholate can induce caspase-dependent apoptosis of cancer cells and reduce tumor growth and metastasis in vivo, and it is safe for normal tissues, with many potential applications in the pharmaceutical industry [66]. In addition, no related biological activities of 3-Oxo-4-phenylbutyronitrile (7.34%) have been reported. To sum up the GC–MS results, the metabolites of strain Y009 are abundant, diverse, and potentially highly valuable. Some identified chemical compounds are well-known for their pharmacological properties based on previous studies, which suggest that the antioxidant and cytotoxic effects demonstrated for Y009 extracts may be due, at least in part, to the presence of these compounds.

Cyclin-dependent protein kinase 2 (CDK2) regulates multiple oncogenic signaling pathways [67]. In this study, the binding affinity of the four leading compounds with CDK2 protein is as follows: 3-Isobutylhexahydropyrrolo[1,2-a]pyrazine-1,4-dione > Ethyl iso-allocholate > 2,5-Piperazinedione,3,6-bis(2-methylpropyl)- > Hexahydropyrrolo[1,2-A]Pyrazine-1,4-Dione. The anticancer potential of the major chemical compound (3-Isobutylhexahydropyrrolo[1,2-a]pyrazine-1,4-dione) was first demonstrated by molecular docking analysis with the CDK2 protein and cytotoxicity assays against three cancer cell lines. This suggests that this compound may be one of the reasons for the broad-spectrum anticancer properties of the Y009 extract. However, 3-Isobutylhexahydropyrrolo[1,2-a]pyrazine-1,4-dione displayed a lower potency than the extract in inhibiting the growth of certain cancer cells. The same phenomenon was also reported in previous research in the field of microbial natural products [20,68]. We, therefore, hypothesized that the combined effect of these components may be the main factor responsible for the antioxidant and potent anticancer properties of Y009 extract. Additionally, it is important to note that the newly isolated mangrove strain has the potential to produce previously unknown bioactive chemicals. It should be noted that mangrove streptomycetes have the potential to synthesize a wide range of bioactive metabolites, and different laboratory culture conditions (pH, temperature, nutrient content, incubation time) may affect the types of secondary metabolites produced (one strain, many compounds, OSMAC) [69]. Overall, this study reveals that *Streptomyces* sp. Y009 is a promising source of bioactive compounds with anticancer and antioxidant activities.

## 5. Conclusions

In summary, the present study revealed that strain Y009, isolated from sediments at a mangrove forest in Guangxi, China, is a potential novel species in the genus *Streptomyces*. This study demonstrated the biopharmaceutical potential of mangrove-derived *Streptomyces* Y009. This strain is an excellent source that is able to produce desirable bioactive compounds with antioxidant and broad-spectrum anticancer properties. Analysis of the lead chemical compound with the cancer therapeutic target CDK2 using docking simulation has also yielded positive results. The significant bioactivity demonstrates that *Streptomyces* sp. Y009 is a rich source of several bioactive compounds. The results suggest that *Streptomyces* sp. strain Y009 may be a promising candidate for further development in the field of chemopreventive drugs. These findings reinforce the evidence of the potential of *Streptomyces* sp. and its ability to produce several bioactive compounds and highlight the important role of exploring microorganisms in mangroves as biological resources. Research, which is currently underway, should further validate and detail the characterization of potential compounds in Y009 metabolites and elucidate the mechanisms underlying the cancer cell death induced by Y009.

## Figures and Tables

**Table 1:** Physiological and biochemical characteristics of *Streptomyces* sp. Y009.

Carbon Source Utilization	Results
D-glucose	+
L-Rhamnose	+
Sucrose	+
D-fructose	+
Lactose	+
L-arabinose	+
Soluble starch	+
Maltose	+
D-mannitol	−
Glycerol	+
D-sorbitol	−
D-xylose	+
Xylitol	+
inulin	+
**Biochemical**	**Results**
NaCl range (%)	0–10
Starch hydrolysis	+
Degradation of cellulose	+
Gelatin liquefaction	+
H_2_S production	−
Production of melanoid pigment	+
Nitrate reduction	+

**Table 2:** The antioxidant activities demonstrated by Y009 extract in both DPPH assay and SOD activity assay.

Concentration of Extract *Streptomyces* sp. Y009 (μg/mL)	Antioxidant Activities	
	Superoxide Dismutase Activity (%)	DPPH Radical Scavenging Activity (%)
75	2.95 ± 0.61	ND
150	14.54 ± 2.48	ND
300	24.69 ± 3.36	1.76 ± 0.37
600	34.71 ± 4.51	14.72 ± 2.65
1200	47.48 ± 1.79	24.30 ± 3.34
BHT (100 μg/mL)	ND	32.16

Data are presented as mean ± standard deviation (*n* = 3); ND, not detected.

**Table 3:** IC_50_ values (μg/mL) of various cell lines at 48 h after adding Y009 extract.

Extract	Cell Line, IC_50_ Values (µg/mL)						
	A549	HepG2	MCF-7	HCT116	CNE	HeLa	L-02
	5.61	8.38	34.99	40.50	42.45	72.15	47.95

**Table 4:** Chemical constituents detected by GC–MS analysis.

No.	Constituents	Retention Time (min)	Molecular Formula	Molecular Weight	Similarity (%)	Area %
1	4-Hydroxy-4-methylpentan-2-one	4.68	C_6_H_12_O_2_	116	95	3.93
2	2-Furanethanol, beta-methoxy-(S)-	7.20	C_7_H_10_O_3_	142	91	1.36
3	Z,Z-2,5-Pentadecadien-1-ol	14.06	C_15_H_28_O	224	90	0.85
4	3-Oxo-4-phenylbutyronitrile	16.53	C_10_H_9_NO	159	95	7.34
5	1,5-dimethyl-1H-indole-3-carbaldehyde	24.53	C_11_H_11_NO	173	90	1.28
6	Hexahydropyrrolo[1,2-A]Pyrazine-1,4-Dione	28.35	C_7_H_10_N_2_O_2_	154	98	9.87
7	2-Methyl-1-hexadecanol	28.7	C_17_H_36_O	257	92	2.96
8	Cyclo(L-Pro-L-Val)	29.30	C_10_H_16_N_2_O_2_	196	87	1.90
9	Hexadecanoic acid, methyl ester	31.26	C_17_H_34_O_2_	270	93	3.50
10	2,5-Piperazinedione,3,6-bis(2-methylpropyl)-	31.60	C_12_H_22_N_2_O_2_	226	96	10.35
11	7,10-Octadecadienoic acid methyl ester	34.41	C_19_H_34_O_2_	295	95	1.57
12	9-Hexadecenoic acid	35.99	C_16_H_30_O_2_	254	89	2.58
13	3-Isobutylhexahydropyrrolo[1,2-a]pyrazine-1,4-dione	36.27	C_11_H_18_N_2_O_2_	210	98	28.32
14	Cyclodecasiloxane, eicosamethyl-	42.50	C_20_H_60_O_10_Si_10_	742	90	1.50
15	Ethyl iso-allocholate	44.15	C_26_H_44_O_5_	437	91	9.48

## Data Availability

The main article and Supplementary Materials have already provided all the data.

## Supplementary Materials

The following supporting information can be downloaded at https://www.mdpi.com/article/10.3390/microorganisms12112300/s1, Table S1. Cultural characteristics of strain Y009 at 28 °C for 14 days; Table S2. Phenotypic properties between strain Y009 and its closely related *Streptomyces*; Table S3: Docking results of four major compounds present in the extract of strain Y009 against CDK protein; Figure S1. a. The relative biomass of Y009 in the pH range of 3.0–11.0. b. The relative biomass of Y009 under gradually increasing salt concentration; Figure S2. Gas chromatography–mass spectrometry (GC–MS) spectrum of the metabolite from strain Y009; Figure S3. The mass spectrum of 3-Isobutylhexahydropyrrolo[1,2-a]pyrazine-1,4-dione identified from the GC–MS analysis of strain Y009.

## References

[1] Soerjomataram I., Bray F. (2021). Planning for tomorrow: Global cancer incidence and the role of prevention 2020–2070. Nat. Rev. Clin. Oncol..

[2] Sung H., Ferlay J., Siegel R.L., Laversanne M., Soerjomataram I., Jemal A., Bray F. (2021). Global cancer statistics 2020: GLOBOCAN estimates of incidence and mortality worldwide for 36 cancers in 185 countries. CA-Cancer J. Clin..

[3] Zheng R., Zhang S., Zeng H., Wang S., Sun K., Chen R., He J. (2022). Cancer incidence and mortality in China, 2016. J. Natl. Cancer Cent..

[4] Steele T.A. (2002). Chemotherapy-induced immunosuppression and reconstitution of immune function. Leuk. Res..

[5] Moxley K.M., Mcmeekin D.S. (2010). Endometrial carcinoma: A review of chemotherapy, drug resistance, and the search for new agents. Oncologist.

[6] Agarwal R., Kaye S.B. (2003). Ovarian cancer: Strategies for overcoming resistance to chemotherapy. Nat. Rev. Cancer.

[7] Hrdý J., Súkeníková L., Petrásková P., Novotná O., Kahoun D., Petříček M., Chroňáková A., Petříčková K. (2020). Inhibition of Pro-Inflammatory Cytokines by Metabolites of Streptomycetes—A Potential Alternative to Current Anti-Inflammatory Drugs?. Microorganisms.

[8] Chin Y.W., Balunas M.J., Chai H.B., Kinghorn A.D. (2006). Drug discovery from natural sources. AAPS J..

[9] Tan L.T.H., Chan C.K., Chan K.G., Pusparajah P., Khan T.M., Ser H.L., Goh B.H. (2019). *Streptomyces* sp. MUM256: A source for apoptosis inducing and cell cycle-arresting bioactive compounds against colon cancer cells. Cancers.

[10] Berdy J. (2005). Bioactive microbial metabolites. J. Antibiot..

[11] Waksman S.A., Schatz A., Reilly H.C. (1946). Metabolism and the chemical nature of *Streptomyces griseus*. J. Bacteriol..

[12] Newman D.J., Cragg G.M. (2016). Natural products as sources of new drugs from 1981 to 2014. J. Nat. Prod..

[13] Sharma S.R., Shah G.S. (2014). Isolation and screening of actinomycetes for bioactive compounds from the marine coast of South-Gujarat Region. Int. J. Res. Sci. Innov..

[14] Lee L.H., Chan K.G., Stach J., Wellington E.M., Goh B.H. (2018). The search for biological active agent(s) from actinobacteria. Front. Microbiol..

[15] Bérdy J. (2012). Thoughts and facts about antibiotics: Where we are now and where we are heading. J. Antibiot..

[16] Hopwood D.A. (1999). Forty years of genetics with *Streptomyces*: From in vivo through in vitro to in silico. Microbiology.

[17] Nobili S., Lippi D., Witort E., Donnini M., Bausi L., Mini E., Capaccioli S. (2009). Natural compounds for cancer treatment and prevention. Pharmacol. Res..

[18] Song Y., Liu G., Li J., Huang H., Zhang X., Zhang H., Ju J. (2015). Cytotoxic and antibacterial angucycline-and prodigiosin-analogues from the deep-sea derived *Streptomyces* sp. SCSIO 11594. Mar. Drugs.

[19] Zhang Z., Yu X., Wang Z., Wu P., Huang J. (2015). Anthracyclines potentiate anti-tumor immunity: A new opportunity for chemoimmunotherapy. Cancer Lett..

[20] Chen C., Ye Y., Wang R., Zhang Y., Wu C., Debnath S.C., Wu M. (2018). *Streptomyces nigra* sp. nov. is a novel actinobacterium isolated from mangrove soil and exerts a potent antitumor activity in vitro. Front. Microbiol..

[21] Du L., Sánchez C., Chen M., Edwards D.J., Shen B. (2000). The biosynthetic gene cluster for the antitumor drug bleomycin from *Streptomyces verticillus* ATCC15003 supporting functional interactions between nonribosomal peptide synthetases and a polyketide synthase. Chem. Biol..

[22] Mangamuri U.K., Muvva V., Poda S., Kamma S. (2012). Isolation, identification and molecular characterization of rare actinomycetes from mangrove ecosystem of Nizampatnam. Malays. J. Microbiol..

[23] Zotchev S.B. (2012). Marine actinomycetes as an emerging resource for the drug development pipelines. J. Biotechnol..

[24] Xu D.B., Ye W.W., Han Y., Deng Z.X., Hong K. (2014). Natural products from mangrove actinomycetes. Mar. Drugs.

[25] Chen J., Xu L., Zhou Y., Han B. (2021). Natural products from actinomycetes associated with marine organisms. Mar. Drugs.

[26] Hu H., Lin H.P., Xie Q., Li L., Xie X.Q., Hong K. (2012). *Streptomyces qinglanensis* sp. nov., isolated from mangrove sediment. Int. J. Syst. Evol. Microbiol..

[27] Ser H.L., Palanisamy U.D., Yin W.F., Abd Malek S.N., Chan K.G., Goh B.H., Lee L.H. (2015). Presence of antioxidative agent, Pyrrolo [1, 2-a] pyrazine-1, 4-dione, hexahydro-in newly isolated *Streptomyces mangrovisoli* sp. nov. Front. Microbiol..

[28] Ser H.L., Palanisamy U.D., Yin W.F., Chan K.G., Goh B.H., Lee L.H. (2016). *Streptomyces malaysiense* sp. nov.: A novel Malaysian mangrove soil actinobacterium with antioxidative activity and cytotoxic potential against human cancer cell lines. Sci. Rep..

[29] Shi S., Cui L., Zhang K., Zeng Q., Li Q., Ma L., Tian X. (2022). *Streptomyces marincola* sp. nov., a novel marine actinomycete, and its biosynthetic potential of bioactive natural products. Front. Microbiol..

[30] Law J.W., Ser H.L., Ab Mutalib N.S., Saokaew S., Duangjai A., Khan T.M., Chan K.G., Goh B.H., Lee L.H. (2019). *Streptomyces monashensis* sp. nov., a novel mangrove soil actinobacterium from East Malaysia with antioxidative potential. Sci. Rep..

[31] Zhu P., Xu Y., Fu J., Liao Y. (2020). *Streptomyces qinzhouensis* sp. nov., a mangrove soil actinobacterium. Int. J. Syst. Evol. Microbiol..

[32] Law J.W.F., Law L.N.S., Letchumanan V., Tan L.T.H., Wong S.H., Chan K.G., Lee L.H. (2020). Anticancer drug discovery from microbial sources: The unique mangrove streptomycetes. Molecules.

[33] Li K., Chen S., Pang X., Cai J., Zhang X., Liu Y., Zhu Y., Zhou X. (2022). Natural products from mangrove sediments-derived microbes: Structural diversity, bioactivities, biosynthesis, and total synthesis. Eur. J. Med. Chem..

[34] Fu P., Yang C., Wang Y., Liu P., Ma Y., Xu L., Su M., Hong K., Zhu W. (2012). Streptocarbazoles A and B, two novel indolocarbazoles from the marine-derived actinomycete strain *Streptomyces* sp. FMA. Org. Lett..

[35] Han Y., Tian E., Xu D., Ma M., Deng Z., Hong K. (2016). Halichoblelide D, a New Elaiophylin Derivative with Potent Cytotoxic Activity from Mangrove-Derived *Streptomyces* sp. 219807. Molecules.

[36] Morisaki H., Nagai S., Ohshima H., Ikemoto E., Kogure K. (1999). The effect of motility and cell-surface polymers on bacterial attachment. Microbiology.

[37] Weisburg W.G., Barns S.M., Pelletier D.A., Lane D.J. (1991). 16S ribosomal DNA amplification for phylogenetic study. J. Bacteriol..

[38] Thompson J.D., Gibson T.J., Plewniak F., Jeanmougin F., Higgins D.G. (1997). The CLUSTAL_X windows interface: Flexible strategies for multiple sequence alignment aided by quality analysis tools. Nucleic Acids Res..

[39] Kumar S., Tamura K., Nei M. (1994). MEGA: Molecular evolutionary genetics analysis software for microcomputers. Bioinformatics.

[40] Kelly K.L., Judd D.B. (1976). Color: Universal Language and Dictionary of Color Names.

[41] Tan L.T.H., Ser H.L., Yin W.F., Chan K.G., Lee L.H., Goh B.H. (2015). Investigation of antioxidative and anticancer potentials of *Streptomyces* sp. MUM256 isolated from Malaysia mangrove soil. Front. Microbiol..

[42] Lee L.H., Zainal N., Azman A.S., Mutalib N.S.A., Hong K., Chan K.G. (2014). *Mumia flava* gen. nov., sp. nov., an actinobacterium of the family Nocardioidaceae. Int. J. Syst. Evol. Microbiol..

[43] Selvin J., Shanmughapriya S., Gandhimathi R., Seghal Kiran G., Rajeetha Ravji T., Natarajaseenivasan K., Hema T.A. (2009). Optimization and production of novel antimicrobial agents from sponge associated marine actinomycetes *Nocardiopsis dassonvillei* MAD08. Appl. Microbiol. Biotechnol..

[44] Mosmann T. (1983). Rapid colorimetric assay for cellular growth and survival: Application to proliferation and cytotoxicity assays. J. Immunol. Methods.

[45] Machado-Carvalho L., Martins T., Aires A., Marques G. (2023). Optimization of Phenolic Compounds Extraction and Antioxidant Activity from Inonotus hispidus Using Ultrasound-Assisted Extraction Technology. Metabolites.

[46] Williams S. (1989). Genus Streptomyces Waksman and Henrici 1943. Bergeys Man. Syst. Bacteriol..

[47] Hong K., Gao A.H., Xie Q.Y., Gao H., Zhuang L., Lin H.P., Ruan J.S. (2009). Actinomycetes for marine drug discovery isolated from mangrove soils and plants in China. Mar. Drugs.

[48] Kim M., Oh H.S., Park S.C., Chun J. (2014). Towards a taxonomic coherence between average nucleotide identity and 16S rRNA gene sequence similarity for species demarcation of prokaryotes. Int. J. Syst. Evol. Microbiol..

[49] Reuter S., Gupta S.C., Chaturvedi M.M., Aggarwal B.B. (2010). Oxidative stress, inflammation, and cancer: How are they linked?. Free Radic. Biol. Med..

[50] Ser H.L., Ab Mutalib N.S., Yin W.F., Chan K.G., Goh B.H., Lee L.H. (2015). Evaluation of antioxidative and cytotoxic activities of *Streptomyces pluripotens* MUSC 137 isolated from mangrove soil in Malaysia. Front. Microbiol..

[51] Law J.W.F., Ser H.L., Duangjai A., Saokaew S., Bukhari S.I., Khan T.M., Lee L.H. (2017). *Streptomyces colonosanans* sp. nov., a novel actinobacterium isolated from Malaysia mangrove soil exhibiting antioxidative activity and cytotoxic potential against human colon cancer cell lines. Front. Microbiol..

[52] Stadtman E.R., Berlett B.S. (1997). Reactive oxygen-mediated protein oxidation in aging and disease. Chem. Res. Toxicol..

[53] Twentyman P.R., Luscombe M. (1987). A study of some variables in a tetrazolium dye (MTT) based assay for cell growth and chemosensitivity. Br. J. Cancer.

[54] Ma A., Jiang K., Chen B., Chen S., Qi X., Lu H., Liu J., Zhou X., Gao T., Li J. (2021). Evaluation of the anticarcinogenic potential of the endophyte, *Streptomyces* sp. LRE541 isolated from *Lilium davidii* var. unicolor (Hoog) Cotton. Microb. Cell Fact..

[55] Almustafa H.I., Yehia R.S. (2023). Antioxidant, Cytotoxic, and DNA Damage Protection Activities of Endophytic Fungus Pestalotiopsis neglecta Isolated from Ziziphus spina-christi Medicinal Plant. Microorganisms.

[56] Wang H., Sun T., Song W., Guo X., Cao P., Xu X., Shen Y., Zhao J. (2020). Taxonomic Characterization and Secondary Metabolite Analysis of NEAU-wh3-1: An Embleya Strain with Antitumor and Antibacterial Activity. Microorganisms.

[57] Yu S., Dong X., Ji H., Yu J., Liu A. (2021). Antitumor activity and immunomodulation mechanism of a novel polysaccharide extracted from *Polygala tenuifolia* Willd. evaluated by S180 cells and S180 tumor-bearing mice. Int. J. Biol. Macromol..

[58] Manimaran M., Gopal J.V., Kannabiran K. (2017). Antibacterial activity of *Streptomyces* sp. VITMK1 isolated from mangrove soil of Pichavaram, Tamil Nadu, India. Proc. Natl. Acad. Sci. Sect. B Biol. Sci..

[59] Rhee K.H. (2002). Isolation and characterization of *Streptomyces* sp. KH-614 producing anti-VRE (vancomycin-resistant enterococci) antibiotics. J. Gen. Appl. Microbiol..

[60] Macherla V.R., Liu J., Bellows C., Teisan S., Nicholson B., Lam K.S., Potts B.C. (2005). Glaciapyrroles A, B, and C, pyrrolosesquiterpenes from a *Streptomyces* sp. isolated from an Alaskan marine sediment. J. Nat. Prod..

[61] Borthwick A.D. (2012). 2,5-Diketopiperazines: Synthesis, reactions, medicinal chemistry, and bioactive natural products. Chem. Rev..

[62] Nicholson B., Lloyd G.K., Miller B.R., Palladino M.A., Kiso Y., Hayashi Y., Neuteboom S.T. (2006). NPI-2358 is a tubulin-depolymerizing agent: In-vitro evidence for activity as a tumor vascular-disrupting agent. Anticancer Drugs.

[63] Mangamuri U.K., Muvva V., Poda S., Manavathi B., Bhujangarao C., Yenamandra V. (2016). Chemical characterization & bioactivity of diketopiperazine derivatives from the mangrove derived pseudonocardia endophytica. Egypt. J. Aquat. Res..

[64] Bhattacharya D., Lai T.K., Saha A., Selvin J., Mukherjee J. (2021). Structural elucidation and antimicrobial activity of a diketopiperazine isolated from a *Bacillus* sp. associated with the marine sponge *Spongia officinalis*. Nat. Prod. Res..

[65] Othman A.R., Abdullah N., Ahmad S., Ismail I.S., Zakaria M.P. (2015). Elucidation of in-vitro anti-inflammatory bioactive compounds isolated from *Jatropha curcas* L. plant root. BMC Complement. Altern. Med..

[66] Thakur R.S., Ahirwar B. (2019). A steroidal derivative from *Trigonella foenum* graecum L. that induces apoptosis in vitro and in vivo. J. Food Drug Anal..

[67] Zhang J., Gan Y., Li H., Yin J., He X., Lin L., Huang W. (2022). Inhibition of the CDK2 and Cyclin A complex leads to autophagic degradation of CDK2 in cancer cells. Nat. Commun..

[68] Goutam J., Sharma G., Tiwari V.K., Mishra A., Kharwar R.N., Ramaraj V., Koch B. (2018). Isolation and Characterization of “Terrein” an Antimicrobial and Antitumor Compound from Endophytic Fungus Aspergillus terreus (JAS-2) Associated from Achyranthus aspera Varanasi, India. Front. Microbiol..

[69] Romano S., Jackson S.A., Patry S., Dobson A.D.W. (2018). Extending the “One Strain Many Compounds” (OSMAC) Principle to Marine Microorganisms. Mar. Drugs.

