# Supplementary material for: Characterization and Bioactive Metabolite Profiling of Streptomyces sp. Y009: A Mangrove-Derived Actinomycetia with Anticancer and Antioxidant Potential
Source: Microorganisms. 2024 Nov 12;12(11):2300. doi: 10.3390/microorganisms12112300 (PMC11596135; doi:10.3390/microorganisms12112300)

Supplementary Material

Article title: Characterization and Bioactive Metabolite Profiling of *Streptomyces* sp. Y009: a Mangrove-Derived *Actinomycetia* With Anticancer and Antioxidant Potential

Journal name: ***Microorganisms***

Authors: Bo Yu [1], Wei Zeng [2] , Yuting Zhou [1], Nan Li [1,*]and Zhiqun Liang [1,*]

[1] State Key Laboratory for Conservation and Utilization of Subtropical Agro-bioresources, Guangxi Microorganism and Enzyme Research Center of Engineering Technology, College of Life Science and Technology, Guangxi University, 100 Daxue Road, Nanning 530004, Guangxi, China;

[2] Key Laboratory of Biochemistry and Molecular Biology (Guilin Medical University), Education Department of Guangxi Zhuang Autonomous Region, School of Intelligent Medicine and Biotechnology, Guilin Medical University, 1 Zhiyuan Road, Guilin 54119, China;

***Correspondence author:**

Nan Li and Zhiqun Liang, Post code: 530004. Tel. / fax: +86 0771 3271181.

E-mail address: linan97690612@163.com (N.L.); zqliang@gxu.edu.cn (Z.L)

Legends of Supplementary Figures and Tables

Supplement Table S1. Cultural characteristics of strain Y009 at 28 ℃ for 14 days

Supplement Table S2. Phenotypic properties between strain Y009 and its closely related *Streptomyces*

Supplement Table S3. Docking results of four major compounds present in the extract of strain Y009 against CDK protein

Supplement Figure S1. a.The relative biomass of Y009 in the pH range of 3.0-11.0. b. The relative biomass of Y009 under gradually increasing salt concentration.

Supplement Figure S2. Gas chromatography–mass spectrometry (GC-MS) spectrum of the metabolite from strain Y009

Supplement Figure S3. The mass spectrum of 3-Isobutylhexahydropyrrolo[1,2-a]pyrazine-1,4-dione identified from the GC-MS analysis of strain Y009

Supplementary Table S1

Agar medium	Aerial mycelium color	Substratemy celium Color	Soluble pigment	Growth
ISP2	Grey-reddish	Brown	None	++
ISP4	White	White grey	None	++
ISP5	Yellow	Light yellowish brown	None	+
ISP7	Grey	Light yellowish brown	Black	+
PDA	Medium gray	Greyish yellow	None	+
Czapek's agar	-	-	-	-
Gauze's agar	White-reddish	Brown	Pink	++
Bennett agar	Purple gray	Brown	None	++
Nutrient agar	Pale green	Light-brown	None	++

++, good growth; +, moderate growth; -, poor growth.

Supplementary Table S2

Characteristic	Y009	*S. thermolilacinus* NBRC 14274^{T*}	*S. fradiae* 319 NBRC 12773^{T**}	*S. somaliensis* DSM 40738^{T***}
**SOURCES**	Sediment	Manures	Soil	Soil
**MORPHOLOGY (ON ISP2)**				
Color of aerial mycelium	Grey-reddish	White	Pink	White
Color of substrate mycelium	Brown	Brown	Yellow	Yellow
Optimum temperature	28 ℃	42 ℃	35 ℃	28 ℃
**CARBON SOURCE UTILIZATION**				
D-glucose	+	+	+	+
Sucrose	+	+	-	+
D-fructose	+	+	+	-
Lactose	+	+	ND	ND
L-arabiose	+	-	+	+
Maltose	+	+	ND	ND
D-mannitol	-	ND	-	-
D-xylose	+	+	+	+
**BIOCHEMICAL**				
Starch hydrolysis	+	-	ND	ND
Degradation of cellulose	+	ND	-	+
H_2S production	-	-	-	ND
Nitrate reduction	+	-	+	ND

+,positive; -, negative. * Date from Talpur et al. (2020). and Jog et al. (2012). **Date from Prakash et al. (2015).
*** Date from Huang et al. (2016).

Supplementary Table S3

S.NO.	Compound Name	Molecular Formula	Molecular Weight (g/mol)	Docking Score Kcal/mol
Comp Ⅰ	3-Isobutylhexahydropyrrolo[1,2-a]pyrazine-1,4-dione	$C_{11}H_{18}N_2O_2$	210	-7.2
Comp Ⅳ	Ethyl iso-allocholate	$C_{26}H_{44}O_5$	437	-6.3
Comp Ⅱ	2,5-Piperazinedione,3,6-bis(2-methylpropyl)-	$C_{12}H_{22}N_2O_2$	226	-6.2
Comp Ⅲ	Hexahydropyrrolo[1,2-A]Pyrazine-1,4-Dione	$C_7H_{10}N_2O_2$	154	-5.7

Supplement Figure S1.

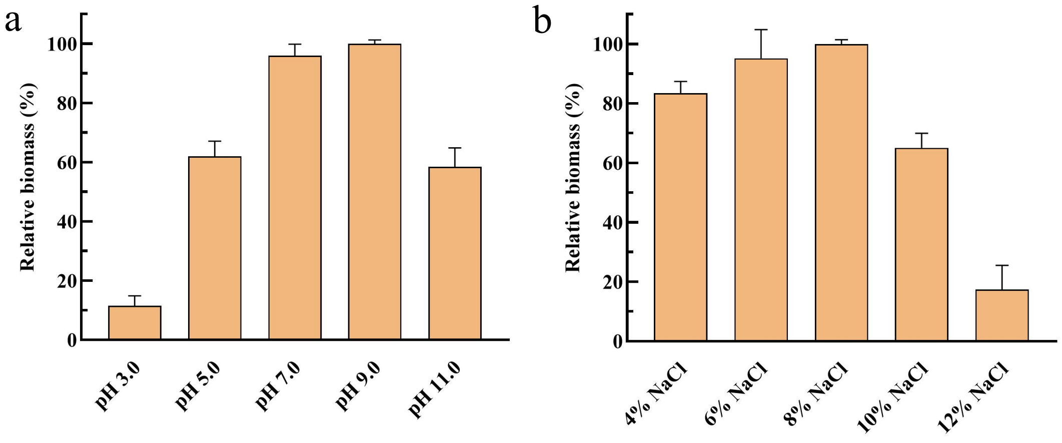

Supplement Figure S2.

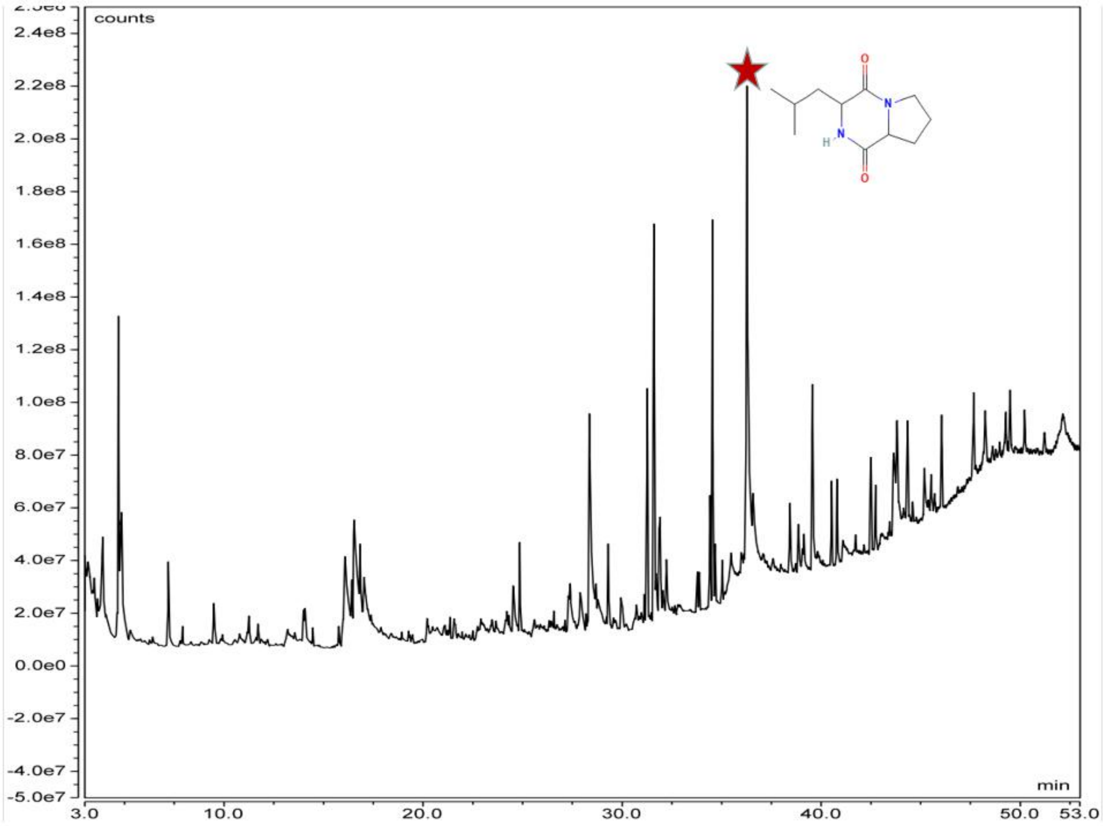

Supplement Figure S3.

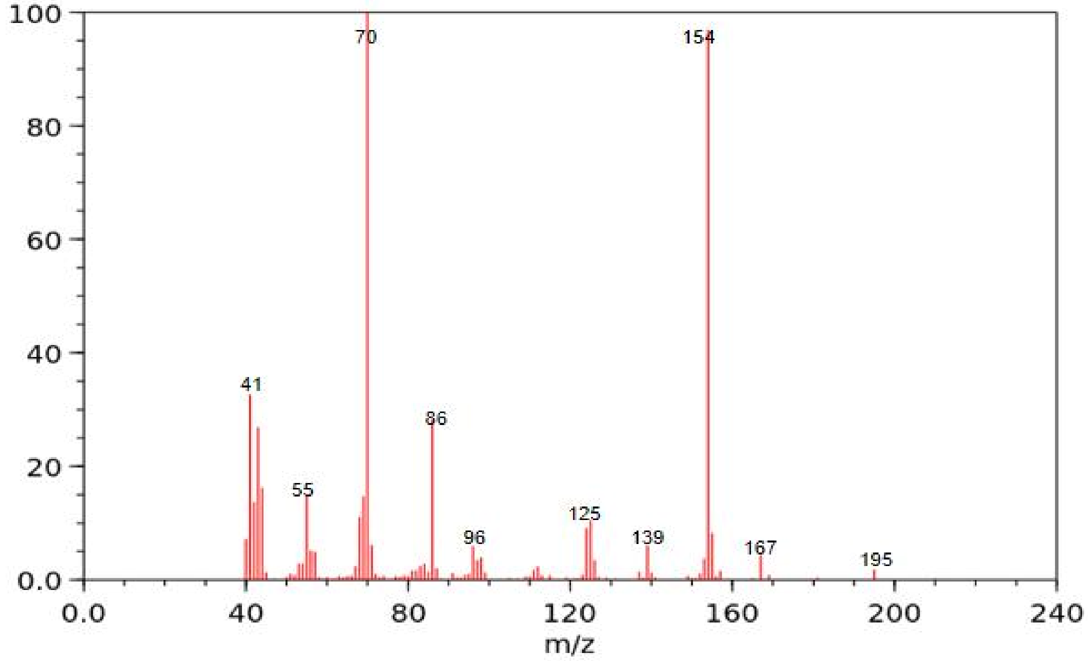

Supplement: Supplementary file 1 [file microorganisms-12-02300-s001.zip › microorganisms-3254936-supplementary.pdf]
